# Autoimmune Gastritis in Children: A Rare Cause of Refractory Iron-Deficiency Anemia

**DOI:** 10.3390/reports9010053

**Published:** 2026-02-04

**Authors:** Alfonso Lendínez-Jurado, Ana García-Ruiz, Manuel Alejandro Sastre-Domínguez, Ana M. Vallejo-Benítez, Andrea Scavarda-Lamberti, Víctor Manuel Navas-López

**Affiliations:** 1 Pediatric Gastroenterology Unit, Vithas Xanit Internacional Hospital, 29620 Málaga, Spain; 2 Distrito Sanitario Málaga-Guadalhorce, 29009 Málaga, Spain; anagaruu@gmail.com; 3 Instituto de Investigación Biomédica de Málaga (IBIMA)-Plataforma BIONAND, 29010 Málaga, Spain; victor.navas@gmail.com; 4 Pediatric Gastroenterology, Hepatology and Nutrition Unit, Reina Sofía Hospital, 14004 Cordoba, Spain; manuelalejandrosastre@gmail.com; 5 Department of Pathology, Regional University Hospital of Malaga, 29010 Málaga, Spain; anavalben@gmail.com; 6 Department of Pathology, Eurofins Clinical Diagnostics, 28007 Madrid, Spain; vbq9@ctes.eurofinseu.com; 7 Pediatric Gastroenterology and Nutrition Unit, Regional University Hospital of Malaga, 29010 Málaga, Spain

**Keywords:** pediatric autoimmune gastritis, refractory iron-deficiency anemia, anti-parietal cell antibodies, atrophic gastritis

## Abstract

**Background and Clinical Significance**: Pediatric autoimmune gastritis (AIG) is a rare and frequently underdiagnosed disorder characterized by chronic immune-mediated inflammation and atrophy of the gastric mucosa. In children, AIG typically presents with iron-deficiency anemia (IDA) refractory to oral iron supplementation, in contrast to the pernicious anemia more commonly observed in adults. Diagnosis relies on a combination of serological markers, such as anti-parietal cell antibodies, and histopathological confirmation, with gastric biopsies demonstrating oxyntic mucosal atrophy and lymphocytic infiltration. Early recognition is essential, particularly in patients with personal or familial autoimmune backgrounds, to prevent long-term complications including nutritional deficiencies and increased gastric neoplasia risk. **Case Presentation**: An 11-year-old boy was referred for evaluation of severe microcytic anemia. He was clinically asymptomatic, with normal growth and physical examination except for mucocutaneous pallor. Celiac disease, thyroid dysfunction, hemoglobinopathies, and infectious or inflammatory gastrointestinal causes were excluded. Despite six months of high-dose oral iron therapy, anemia persisted. Upper gastrointestinal endoscopy showed macroscopically normal mucosa; however, histopathological analysis of gastric body biopsies revealed chronic atrophic gastritis. Serological testing confirmed autoimmune etiology, with positive anti-parietal cell antibodies and hypergastrinemia. Since diagnosis, the patient has required two courses of intravenous iron supplementation, and remains under close follow-up for associated autoimmune and hematologic conditions. **Conclusions**: Refractory IDA may represent the sole clinical manifestation of AIG in pediatric patients, even in the absence of gastrointestinal symptoms. Histological assessment is crucial, as endoscopic findings may be normal. Early diagnostic suspicion allows timely management focused on correction of nutritional deficiencies and long-term surveillance to mitigate neoplastic risk. AIG should therefore be considered in children with anemia unresponsive to conventional iron therapy.

## 1. Introduction and Clinical Significance

Anemia is one of the most common hematologic disorders in childhood, with iron-deficiency anemia (IDA) being its leading cause worldwide [1]. In pediatric patients, IDA is most often related to inadequate dietary intake, increased physiological requirements, or chronic blood loss, and it typically responds to appropriately dosed oral iron therapy [1]. Consequently, IDA that is refractory to adequate oral iron supplementation warrants further evaluation, as it may reflect an underlying inflammatory, systemic, or gastrointestinal disorder [2].

Pediatric autoimmune gastritis (AIG) is a rare condition characterized by chronic inflammation and atrophy of the gastric mucosa, predominantly affecting the corpus and fundus. It is mediated by autoantibodies directed against parietal cells and, occasionally, intrinsic factor. Although its prevalence in children is low, AIG is likely underdiagnosed due to non-specific symptoms and a low index of clinical suspicion [3,4,5]. To date, no population-based pediatric studies have established a true annual incidence, and available data derive mainly from retrospective series and multicenter cohorts, confirming that AIG represents only a small minority of chronic gastritis cases diagnosed in childhood [3,4,5].

The clinical presentation in children is typically dominated by IDA refractory to oral iron therapy, which represents the most common manifestation in this age group. This contrasts with the adult presentation, where pernicious anemia is more prevalent [3,6,7,8]. Gastrointestinal symptoms are often non-specific or absent; however, some patients may report epigastric pain, dyspepsia, or symptoms related to vitamin B12 deficiency in advanced stages [3,9]. A common association with other autoimmune diseases is well-documented, including type 1 diabetes mellitus and autoimmune thyroiditis. Less frequently, associations with Th2-type allergic diseases, such as atopic dermatitis, rhinitis, and asthma, have been observed [4,5,9].

In the pediatric population, AIG is most commonly diagnosed in late childhood or adolescence. The largest international multicenter study reported a median age at diagnosis between 12 and 14 years, with the majority of patients diagnosed during adolescence [4]. These findings are consistent with other multicenter and single-center series reporting diagnosis typically between 10 and 16 years of age [3,5,6]. Younger children can be affected, although they are less frequently represented in published cohorts [5,7].

The diagnosis relies on a combination of serological findings (anti-parietal cell and/or anti-intrinsic factor antibodies), laboratory tests (hypergastrinemia, anemia, iron deficiency), and histological confirmation via gastric biopsy. Characteristic histopathological features include oxyntic mucosal atrophy, lymphocytic infiltration, intestinal or pseudopyloric metaplasia, and frequently, enterochromaffin-like (ECL) cell hyperplasia [3,4,5,6,7,10]. *Helicobacter pylori* infection does not appear to be implicated in the pathogenesis of most pediatric cases, although it may coexist without significantly altering the disease course [3,4,6,8].

In clinical practice, it is important to distinguish AIG from *H. pylori*–related atrophic gastritis in children. AIG is characterized by corpus-predominant atrophy, autoimmune serological markers (anti-parietal cell and/or anti-intrinsic factor antibodies), and hypergastrinemia, and should be suspected in cases of refractory IDA or in patients with autoimmune comorbidities. In contrast, *H. pylori*-associated gastritis typically presents with antral-predominant or pangastric inflammation driven by chronic infection, and atrophy—when present—may improve after eradication therapy [3,4,6,8].

Potential long-term complications include the progression of intestinal metaplasia, ECL cell hyperplasia, and, in rare instances, the development of type 1 gastric neuroendocrine tumors (NETs) or adenocarcinoma. These risks justify the recommendation for periodic endoscopic surveillance [3,4,10,11]. While specific pediatric guidelines are lacking, endoscopic monitoring and management of nutritional deficiencies are generally advised [3,11].

Treatment is primarily focused on correcting anemia and micronutrient deficiencies (iron and vitamin B12), typically through oral or parenteral supplementation based on patient tolerance and response [8,12]. Current evidence does not support the reversal of histological damage with available treatments, and the disease typically follows a chronic and progressive course [3,8,13]. For patients with concurrent autoimmune conditions, a comprehensive evaluation and multidisciplinary follow-up are recommended [4,5,9].

In summary, AIG should be suspected in cases of refractory IDA, particularly in children with a personal or family history of autoimmune diseases. Diagnosis requires both serological and histopathological confirmation. Management is based on nutritional supplementation and endoscopic surveillance to prevent neoplastic complications. We report a case of a pediatric patient with IDA refractory to oral supplementation, focusing on the clinical progression and diagnostic workup that led to the final diagnosis.

## 2. Case Presentation

An 11-year-old male patient, with no relevant personal medical history, was referred for evaluation following the incidental discovery of IDA (Hemoglobin 8.1 g/dL, Hematocrit 26.2%, Mean Corpuscular Volume 53 fL, Mean Corpuscular Hemoglobin 16.2 pg, Platelets 310 × 10^3^/µL, Iron 13 µg/dL, Ferritin 6 ng/mL). His personal medical history was unremarkable. A notable family history included maternal *Helicobacter pylori* gastritis four years prior.

The patient reported no reflux, vomiting, diarrhea or other gastrointestinal symptoms. His physical and neurodevelopmental growth were adequate, and he denied asthenia. Dietary history revealed a varied diet including all food groups, with daily dairy intake (1–2 glasses of milk). Following the anemia diagnosis, the family increased his consumption of iron-rich foods combined with citrus fruits.

Physical examination revealed mild generalized mucocutaneous pallor, with no abdominal masses or hepatomegaly. Cardiorespiratory auscultation was unremarkable, with no audible murmurs.

Initial laboratory work-up ruled out celiac disease (Immunoglobulin A 452 mg/dL, anti-Tissue Transglutaminase IgA antibodies 2.27 U/mL [cut-off < 5]) and thyroid dysfunction (TSH 1.67 mU/L, T4 6.4 µg/dL). A beta-thalassemia screen and a hemoglobinopathy screen were negative, with Hemoglobin A2 at 2.1% (reference range 1.5–3.5%) and Hemoglobin F at 0.9% (reference range 0.0–2.0%). Lipid, renal, and hepatic profiles were within normal limits, with a mild elevation of the erythrocyte sedimentation rate (27 mm; cut-off < 15) and no increase in C-reactive protein. Fecal calprotectin levels were elevated at 275 µg/g (cut-off < 50), while fecal occult blood testing, stool culture, and ova and parasite examinations were negative.

Oral iron therapy with sucrosomial iron at 1.3 mg/kg/day (42 mg/day) was initiated for a three-month period and was well tolerated, with good adherence and no reported gastrointestinal adverse effects. Due to an incomplete biochemical and hematological response, the dose was subsequently increased to 1.8 mg/kg/day (60 mg/day) for an additional three months, with similarly good tolerability and adherence. Despite six months of oral iron supplementation, iron parameters and anemia failed to correct completely.

**Figure 1:**
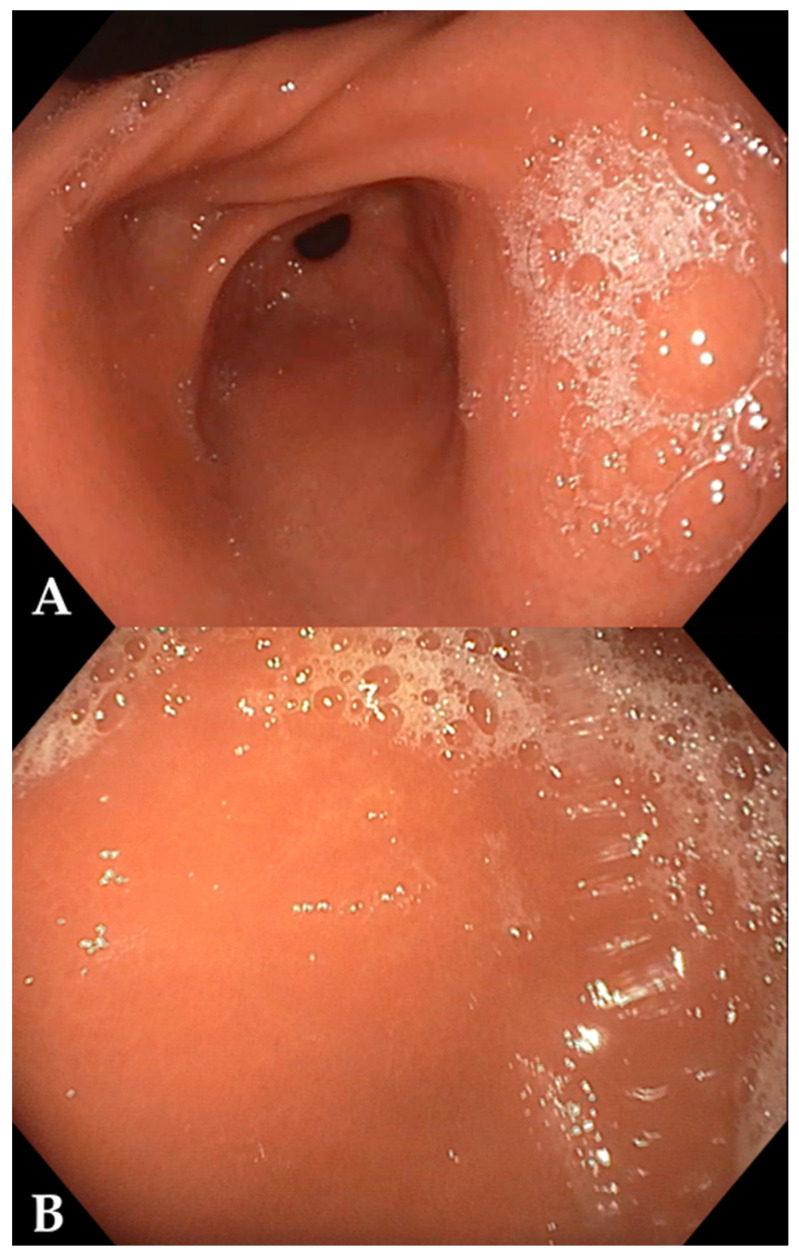
Endoscopic findings on upper gastrointestinal endoscopy. The mucosa appears endoscopically preserved in both antrum (**A**) and gastric body (**B**). This macroscopic normality is a hallmark of the initial phases of autoimmune gastritis, where the immune-mediated mucosal damage is only detectable at the microscopic level.

Given the refractoriness to oral iron therapy and the suspicion of a malabsorptive pathology, an upper and lower GI endoscopy was scheduled. The procedure revealed no macroscopic lesions (Figure 1). Biopsies were taken from the duodenum, gastric antrum/incisura, gastric body and esophagus, and a colonic mapping was also performed.

**Figure 2:**
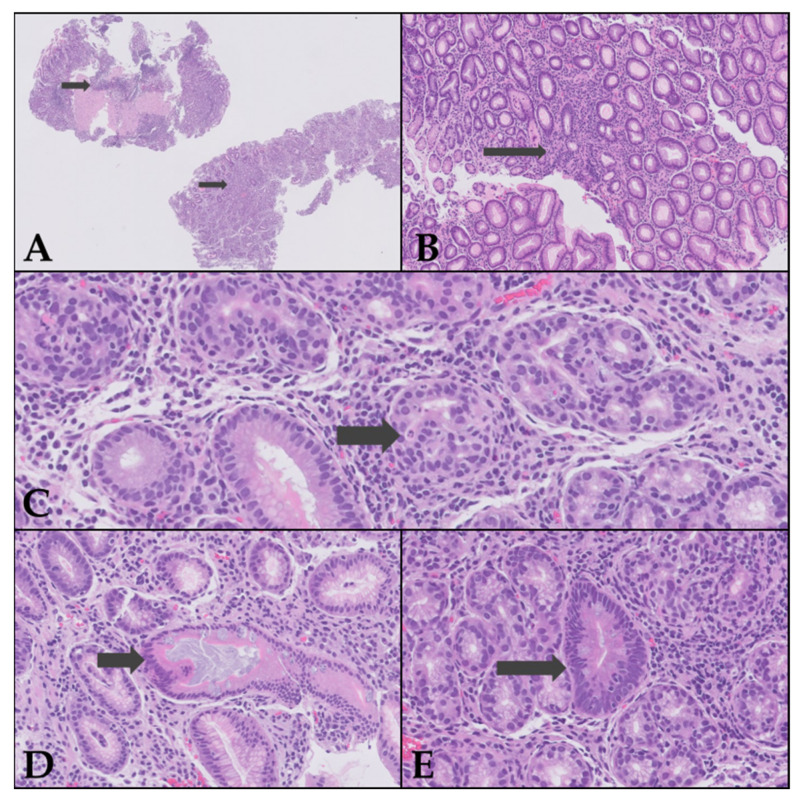
Histological features of chronic atrophic gastritis (H&E stain). (**A**) Atrophic mucosal fragments with focal intestinal metaplasia and chronic lymphoplasmacytic inflammation (×5). (**B**) Gastric mucosa with mild-to-moderate oxyntic gland atrophy, loss of parietal cell profile, and dense lymphoplasmacytic infiltrate within the lamina propria (×2). (**C**) Intense lymphocytic infiltrate cuffing residual oxyntic glands with focal glandular destruction, consistent with marked focal atrophy (×20). (**D**) Residual mucous cell–predominant glands with irregular luminal architecture, accompanied by chronic inflammatory infiltrate (×10). (**E**) Advanced intestinal metaplasia with goblet cells and columnar epithelium of intestinal phenotype; mild stromal fibrosis with persistent lymphoplasmacytic infiltrate (×10). A complementary study using Warthin–Starry (W-S) staining and serial sectioning of the specimen was performed twicex; no bacillary structures suggestive of *Helicobacter pylori* were identified.

Histopathological examination of the biopsies revealed findings consistent with chronic atrophic gastritis of the gastric body (Figure 2), while duodenal/ileal and colonic biopsies were histologically normal. Based on these results, further serological tests were requested. The results showed normal vitamin B12 levels (918 pg/mL, reference 220–950 pg/mL), negative anti-intrinsic factor antibodies, positive anti-parietal cell antibodies (>169 U/mL), reduced levels of pepsinogen I (9.10 ng/mL, reference range 25–200) and hypergastrinemia (271.00 pg/mL, cut-off < 100), confirming the diagnosis of autoimmune gastritis.

Following confirmation of AIG, clinical follow-up focused on the management of IDA while the patient continued oral sucrosomial iron with good tolerability and adherence (co-administered with citrus foods and separated from calcium-containing products). Despite a progressive improvement in hemoglobin levels and red cell indices, iron stores remained depleted and transferrin saturation persistently low, accompanied by clinically relevant symptoms of asthenia and fatigue. In this context—combining biochemical iron depletion with a symptomatic burden—the decision was made to escalate treatment to intravenous iron therapy.

After the first intravenous iron infusion, a clear and clinically meaningful improvement was observed, with normalization of iron metabolism parameters and a parallel resolution of the patient’s asthenic symptoms. However, over the subsequent weeks, both laboratory and clinical relapse occurred, characterized by a renewed decline in ferritin and transferrin saturation together with recurrence of fatigue. Given this pattern of transient response and recurrent iron depletion, a second intravenous iron infusion was therefore scheduled as part of the ongoing management strategy.

**Figure 3:**
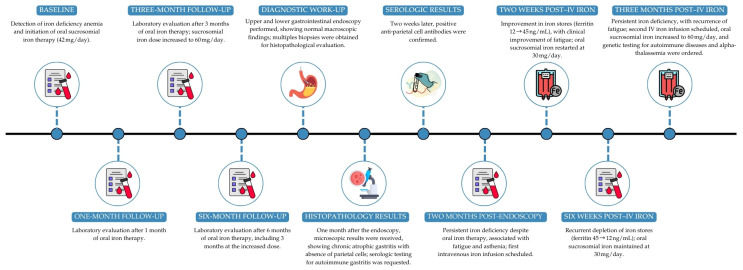
Clinical timeline of the reported case.

This clinical course—from the initial histopathological diagnosis of AIG, through oral iron supplementation and escalation to intravenous iron due to symptomatic iron-store depletion, followed by an initial response and subsequent biochemical and clinical relapse—is summarized chronologically in the timeline figure (Figure 3). The corresponding longitudinal trends in hematologic and iron metabolism parameters are detailed in Supplementary Table S1 (Table S1).

To support etiological clarification and long-term management planning, the diagnostic evaluation was expanded to include extended genetic testing for autoimmune disorders and molecular analysis to exclude α-thalassemia as a contributor to the persistent microcytosis and altered iron profile.

## 3. Discussion

The case of a pediatric patient with refractory IDA and a subsequent diagnosis of AIG presents several clinical and management challenges. The pathophysiology of anemia in this context is related to oxyntic mucosal atrophy, which reduces gastric acid secretion and compromises iron absorption, even in the presence of an adequate diet and without absorption inhibitors such as calcium or tannins [8]. The literature indicates that IDA in AIG is often persistent and poorly responsive to oral iron, frequently requiring intravenous supplementation [3,9,10]. This pattern—poor response to oral iron and the need for intermittent intravenous iron—aligns with contemporary pediatric series describing recurrent iron-store depletion despite adherence to therapy [3,8,10].

The differential diagnosis should include common causes of anemia in pediatrics, such as hemoglobinopathies, celiac disease, and nutritional deficiencies, but also less frequent pathologies like AIG, particularly in cases of treatment refractoriness and a family history of autoimmunity [9,11]. The American Gastroenterological Association recommends performing an upper gastrointestinal endoscopy in cases of unexplained anemia, regardless of the presence of gastrointestinal symptoms, to rule out structural lesions and confirm the histological diagnosis [4,6]. Our approach followed this pathway, prioritizing exclusion of common etiologies and proceeding to endoscopic assessment when IDA remained unexplained and refractory.

In the present case, the absence of digestive symptoms and adequate growth complicated the clinical suspicion, which aligns with pediatric case series where the presentation is insidious and anemia is the primary finding [3,12,13]. The exclusion of *Helicobacter pylori* is relevant, as although it can be associated with IDA in children, significant gastric atrophy is rare in this population, and most AIG cases are *H. pylori*-negative [5,12,13]. Consistent with published pediatric cohorts, our patient’s endoscopy was macroscopically normal and the diagnosis relied on corpus-predominant histological changes, a common scenario in children where endoscopic appearance lacks sensitivity [3,5,12,13]. Age at diagnosis in pediatric series ranges from school age to adolescence, with microcytic anemia often severe at presentation; our patient, diagnosed at 11 years with marked microcytosis and low transferrin saturation, fits this spectrum [3,4,5,12,13]. Histologically, pediatric AIG typically shows oxyntic mucosal atrophy with lymphoplasmacytic infiltration and can exhibit early endocrine changes (e.g., ECL-cell hyperplasia), even when endoscopy is unremarkable—features that parallel our findings [5,10,13].

The association with other autoimmune diseases, such as thyroiditis and type 1 diabetes, is frequent and justifies the systematic screening for comorbidities in these patients [3,12,13]. Furthermore, an association with Th2-type allergic diseases and eosinophilic gastritis has been described, which broadens the clinical spectrum and necessitates a multidisciplinary approach [14,15]. Serologically, anti-parietal cell antibodies are the most frequent finding at pediatric onset, whereas intrinsic factor antibodies are less consistently detected in children compared with adults; hypergastrinemia is common and may precede vitamin B12 deficiency, which often emerges later in the disease course [3,5,12,13]. Given the persistent microcytosis and iron-handling profile, we also pursued molecular testing to exclude concurrent α-thalassemia, as recommended in pediatric algorithms addressing early-onset or refractory cases [15].

Management should focus on correcting nutritional deficiencies, with periodic monitoring of iron and vitamin B12 levels, and on endoscopic surveillance to detect histological progression, intestinal metaplasia, and the risk of gastric neoplasia [3,5,7,14,15]. The European Society of Gastrointestinal Endoscopy suggests surveillance every 3–5 years, although the evidence is limited [6]. There is no evidence of histological reversal with treatment, making long-term follow-up essential [3,10]. Because pediatric-specific surveillance data are scarce, current practice extrapolates from adult guidance; at present, the patient remains under active follow-up, and we are still within the work-up phase for potentially associated conditions. In light of the limited pediatric-specific evidence to support fixed surveillance intervals, we will not set a date for repeat endoscopy at this time. Instead, endoscopic reassessment will be scheduled once we have the results of the pending studies and in accordance with the patient’s clinical course and serial laboratory data (hemoglobin, indices, ferritin, transferrin saturation, vitamin B12, and gastrin) [6,11,15]. Concurrently, periodic screening for associated autoimmunity (thyroid function/antibodies, type 1 diabetes markers) will continue as part of ongoing surveillance [3,6,12,15].

As limitations, this is a single-case report with limited external generalizability; causality between interventions and outcomes cannot be established, and pediatric surveillance intervals are extrapolated from adult recommendations due to scarce child-specific data, underscoring the need for prospective pediatric studies

## 4. Conclusions

Refractory IDA in pediatrics should be considered a marker for AIG, especially in the context of a family history of autoimmunity. Early diagnosis and multidisciplinary management are crucial for preventing nutritional and neoplastic complications, thereby optimizing the patient’s prognosis and quality of life. In practical terms, any child with IDA unresponsive to optimized oral iron—particularly when endoscopy is macroscopically normal—should undergo targeted evaluation for AIG, with histological confirmation guiding iron repletion (including intravenous iron when needed) and individualized surveillance. In our case, the patient remains under active follow-up while the work-up for associated autoimmune conditions is being completed, and future endoscopic reassessment will be scheduled based on clinical evolution and serial laboratory results rather than a fixed interval, given the current paucity of pediatric-specific evidence.

## Data Availability

The original data presented in the study are included in the article; further inquiries can be directed to the corresponding author.

## Supplementary Materials

The following supporting information can be downloaded at: https://www.mdpi.com/article/10.3390/reports9010053/s1, Table S1: Hematologic and iron metabolism parameters across timepoints.

## Abbreviations

The following abbreviations are used in this manuscript:

IDA	Iron-deficiency anemia
AIG	Autoimmune gastritis
ECL	Enterochromaffin-like
NET	Neuroendocrine tumor
TSH	Thyroid-Stimulating hormone
GI	Gastrointestinal
H&E	Hematoxylin and eosin
W-S	Warthin–Starry
