# Supplementary material for: Autoimmune Gastritis in Children: A Rare Cause of Refractory Iron-Deficiency Anemia
Source: Reports (MDPI). 2026 Feb 4;9(1):53. doi: 10.3390/reports9010053 (PMC12921746; doi:10.3390/reports9010053)
Supplement: Supplementary file 1 [file reports-09-00053-s001.zip › Table S1.pdf]

Table S1. **Hematologic and iron metabolism parameters across timepoints.**

Parameter (units)	Baseline	1 Month after Oral Iron Therapy	3 Months after Oral Iron Therapy	6 Months after Oral Iron Therapy	1 Month Post-Endoscopy	2 Months Post-Endoscopy	2 Weeks after First IV Iron Infusion	6 Weeks after First IV Iron Infusion	2 Weeks before Second IV Iron Infusion
Hemoglobin (g/dL)	8.1	8.9	10.2	11.5	11.4	12.8	12.5	13.2	13.1
Hematocrit (%)	26.2	36	37	40	36.8	41			
MCV (fL)	53	63.2	61.1	64.1	65.9	68.7	68.5	70.2	72.7
MCH (pg)	16.2	15.8	16.8	18.6	20.4	21.5	21.8	23.2	23
RDW (%)	20.1	22.9	23.1	24	18.4	18.2	17.3	16.1	14.7
Platelets (x10^3/µL)	310	430	516	439	410	365	342	291	286
Reticulocytes (%)	—	1.37	0.67	1.01	—	0.97	—	—	—
Reticulocytes (x10^6/µL)	—	0.0774	0.0406	0.0625	—	0.0576	—	—	—
Serum iron (µg/dL)	13	24	15	35	28	68	42	36	34
Ferritin (ng/mL)	6	6	7	10	13.6	12	45	12	11
Transferrin (mg/dL)	—	385	407	409	341	401	328	317	304
Transferrin saturation (%)	—	5	3	7	6	13	9.1	7.9	8.5
TIBC (µg/dL)	—	489	517	519	—	509	—	—	—
Vitamin B12 (pg/mL)	—	949	1008	804	918	931	667	—	721
